# Exposing kinetic disparities between inflammasome readouts using time-resolved analysis

**DOI:** 10.1016/j.heliyon.2024.e32023

**Published:** 2024-05-29

**Authors:** Matthew Herring, Alexander Persson, Ryan Potter, Roger Karlsson, Eva Särndahl, Mikael Ejdebäck

**Affiliations:** a School of Medical Sciences, Faculty of Medicine and Health, Örebro University, Örebro, Sweden; b Inflammatory Response and Infection Susceptibility Centre (iRiSC), Örebro University, Örebro, Sweden; c School of Bioscience, Systems Biology Research Centre, University of Skövde, Skövde, Sweden; d Department of Clinical Neuroscience, Institute of Neuroscience and Physiology, Sahlgrenska Academy, Göteborg University, Göteborg, Sweden; e Nanoxis Consulting AB, Göteborg, Sweden; f Department of Infectious Diseases, Institute of Biomedicine, Sahlgrenska Academy, Göteborg University, Göteborg, Sweden; g Department of Clinical Microbiology, Sahlgrenska University Hospital, Region Västra Götaland, Göteborg, Sweden

**Keywords:** NLRP3 inflammasome, ASC-Specks, Cell response, THP-1 cells, Human macrophages, Cytokines, Live-cell imaging, LDH leakage

## Abstract

The NLRP3 inflammasome is an intracellular multiprotein complex described to be involved in both an effective host response to infectious agents and various diseases. Investigation into the NLRP3 inflammasome has been extensive in the past two decades, and often revolves around the analysis of a few specific readouts, including ASC-speck formation, caspase-1 cleavage or activation, and cleavage and release of IL-1β and/or IL-18. Quantification of these readouts is commonly undertaken as an endpoint analysis, where the presence of each positive outcome is assessed independently of the others. In this study, we apply time-resolved analysis of a human macrophage model (differentiated THP-1-ASC-GFP cells) to commonly accessible methods. This approach yields the additional quantifiable metrics time-resolved absolute change and acceleration, allowing comparisons between readouts. Using this methodological approach, we reveal (potential) discrepancies between inflammasome-related readouts that otherwise might go undiscovered. The study highlights the importance of time-resolved data in general and may be further extended as well as incorporated into other areas of research.

## 1 Introduction

Inflammasomes are large, intracellular multiprotein complexes formed in several cell types in response to inflammatory stimuli [1]. Inflammasome composition facilitates proximity-activated cleavage of pro-caspase-1 into active caspase-1, which further allows subsequent processing of inactive pro-interleukins (IL)-1β and IL-18 into their biologically active forms.

The most extensively studied inflammasome is the nod-like receptor family pyrin domain containing 3 (NLRP3) inflammasome. In cellular experiments, NLRP3 inflammasome activation is often determined by measuring presence of the apoptosis-associated speck-like protein containing a card (ASC)-speck [[2], [3], [4]], caspase-1 cleavage/activation [[4], [5], [6]], and IL-1β/18 maturation and release [4,6]. Often, such analyses are performed at a single or a few time points following cellular stimulation in order to state whether the stimulation in question results in activation of the inflammasome. Presence of ASC-specks is commonly used as a marker of inflammasome formation and can be detected by fluorescence microscopy [2,3], flow cytometry or Western blot [3,4]. Caspase-1 activation, a hallmark of inflammasome activation, can be assessed by fluorescence microscopy using fluorochrome-labeled inhibitors of caspases (FLICA) assays [4,7], or inferred by caspase-1 cleavage that can be detected by Western blot [5,6]. In addition to cleaving pro-IL-1β and pro-IL-18, caspase-1 also cleaves gasdermin D (GSDMD). The *N*-terminal fragment of GSDMD is responsible for membrane poration and, by that, cytokine release [[8], [9], [10]].The cytokines can be quantified by ELISA or a similar method. Western blot can be further utilized to determine the ratios of uncleaved/cleaved IL-1β and IL-18 in order to estimate caspase-1 activity. The final step of experimental NLRP3 inflammasome activation is pyroptosis, a proinflammatory, caspase-1 mediated, GSDMD-dependent cell death [8]. Pyroptosis, being a cell lytic event, is typically assessed via presence of lactate dehydrogenase (LDH) [11,12] in the extracellular media. All of the above methods are commercially available and readily accessible in most labs.

While these readouts are commonly used to verify inflammasome activity, the temporal association between them is not fully understood. Although the sequence of ASC-speck formation, followed by caspase-1 activation, cleavage of IL-1β, IL-18 and GSDMD, GSDMD pore formation, and cytokine release, has been established [13], several questions remain. For example, the trigger-specific time-kinetics or amplitudes of inflammasome response as well as the connection between pore formation by GSDMD and cell rupture leading to the release of LDH are events that remain to be elucidated. Further, there is still some debate whether or not the ASC-speck is the site of inflammasome function [14], which opens up the question of what constitutes “an inflammasome” and consequently which readout(s) that best reflect inflammasome function. Time-resolved analysis can highlight discrepancies in readout kinetics, which may guide investigation of the mechanism behind, and connection between, the end readouts.

Inflammasome activity can be investigated using several cell models, including murine bone marrow-derived macrophages, human peripheral blood mononuclear cells, and established cell culture models. The THP-1 cell culture model has been extensively used in inflammasome research [[15], [16], [17], [18], [19]] and, when differentiated, provides a homogenous model that approximates human macrophage function and behaviour [20]. Here we use a differentiated THP-1-ASC-GFP cell culture model to illustrate the time-resolved dynamics of inflammasome readouts by combining analysis of ASC-speck formation, extracellular IL-1β/IL-18 and LDH leakage. By doing so, a dynamic cell response over inflammasome activity kinetics mediated by common inflammasome triggers can be described.

## 2 Results and discussion

### 2.1 ASC-speck dynamics differ between triggers

As a readout for inflammasome formation, ASC-speck formation is often measured as an endpoint analysis. In order to provide time-resolved data of ASC-speck formation spanning the full duration of speck formation, the number of ASC-GFP-specks in PMA-differentiated and LPS-primed THP-1-ASC-GFP cell populations activated with either ATP, MSU or nigericin was quantified at multiple time points using fluorescence microscopy. The used concentrations of ATP [14,[21], [22], [23], [24], [25], [26], [27], [28], [29], [30]], MSU [[31], [32], [33], [34], [35], [36]] and nigericin [21,22,25,27,[37], [38], [39], [40]] are commonly found in the literature for NLRP3 activation in THP-1 and other cell models. Untreated controls were also imaged to investigate any potential effects of imaging (Fig. S1), which was found to have no impact on ASC-speck formation.

**Fig. 1:**
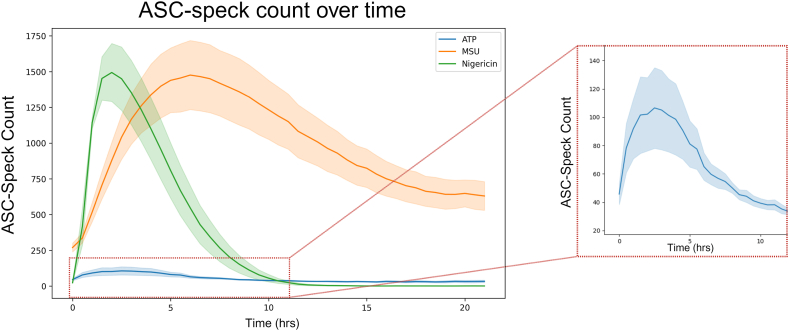
ASC-speck dynamics differ between triggers. ASC-speck count in PMA-differentiated, LPS-primed THP-1 cells after triggering with ATP, MSU or nigericin. ASC-GFP specks were imaged every 30 min for 21 h by fluorescence microscopy and automatically quantified using the Weka segmentation plugin for ImageJ. Inset shows ATP triggered ASC-speck counts during the first 11 h. Data are represented as mean (solid line) ± SEM (shaded area), n = 5. Created with BioRender.com.

ASC-speck formation as a readout for inflammasome formation is often measured as an endpoint analysis, which for ATP, is commonly measured up to 1 h post activation [14,41,42]. The approach of addressing single time points risks missing the window time point at which differences are largest, thereby underestimating the magnitude of the result as well as providing no information on the duration of the cellular event. While we see an increase in ASC-speck formation at 1 h, the highest number of ASC-specks is formed at 3 h post ATP activation (Fig. 1 and inset). Likewise, nigericin-activated cells are commonly analyzed between 30 min and 1 h post activation [[38], [39], [40],[43], [44], [45]]. Our data suggests that, with regards to ASC-speck formation, occurring at 30 min and lasting up to 5 h post nigericin activation (Fig. 1), it may be beneficial to evaluate additional time points. ASC-speck formation triggered by MSU however, is frequently assessed 12 h or more after activation [32,44,46,47]. Based on our data, assessment at these time points would miss the peak ASC-speck formation at 6 h as well as the duration (Fig. 1).

### 2.2 Time-resolved analysis allows for analysis of additional metrics

To quantify the number of formed ASC-specks *per se* does not provide information on when they were formed, and ASC-speck fluorescence does not remain indefinitely. Despite the homogeneity of THP-1 cells, time-resolved data reveal discrepancies between individual cells (Video S1–S3). Since an endpoint analysis will only show the cumulative number of specks formed, time-resolved data allows for assessment of the time at which the speck formation is increasing the most. By combining automated ASC-GFP-speck quantification to images obtained by fluorescence microscopy, we evaluated the absolute change in ASC-speck formation (Fig. 2A–C) to determine between which time points the number of ASC-specks is increasing the most in the cell population. Even though the time of peak formation differed with trigger (Fig. 1), our data revealed that the time at which absolute change increased the most was during the first hour of activation regardless of trigger tested (Fig. 2A–C).

**Fig. 2:**
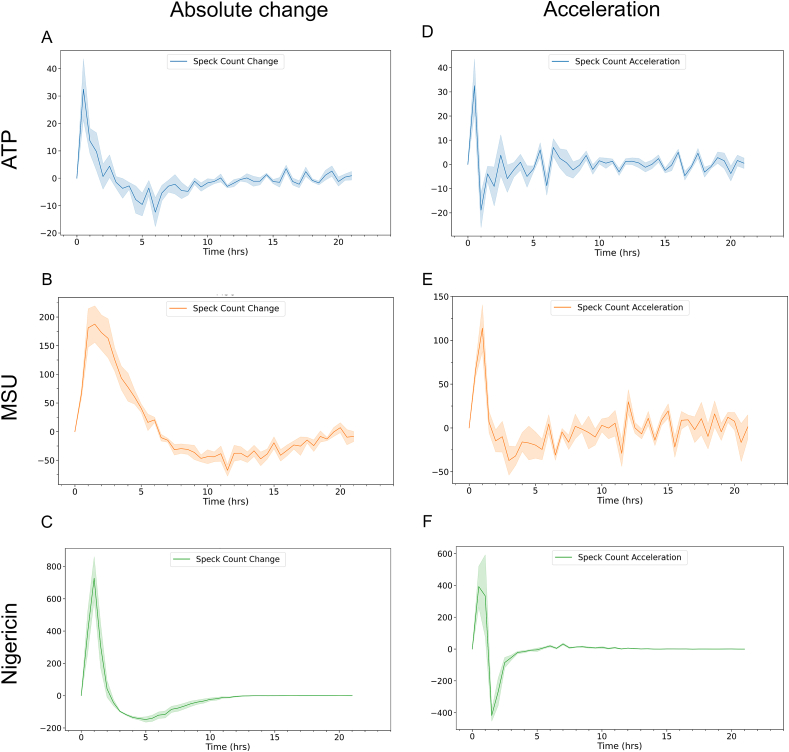
Time-resolved analysis allows for analysis of additional metrics. Quantifiable metrics of ASC-speck formation obtained by time-resolved analysis in PMA-differentiated, LPS-primed THP-1 cells. Absolute change (A–C) in ASC-speck number after triggering with A) ATP, B) MSU or C) nigericin and acceleration (D–F) of ASC-speck formation after triggering with D) ATP, E) MSU or F) nigericin. ASC-GFP specks were imaged every hour for 21 h by fluorescence microscopy and automatically quantified using the Weka segmentation plugin for ImageJ. Data is shown as mean (solid line) ± SEM (shaded area), n = 5. Created with BioRender.com.

Supplementary data related to this article can be found online at https://doi.org/10.1016/j.heliyon.2024.e32023

The following are the Supplementary data related to this article:

video 1 video 1

video 2 video 2

video 3 video 3

Acceleration is defined as the rate of change of velocity or rate per unit of time. In a biological context, this indicates how fast a process is changing, providing a quantifiable metric regarding a system's ability to regulate the process being evaluated. Values around zero indicate no change, while deviation from zero in either direction indicates a possible change in the process. Importantly, a decrease in acceleration does not *per se* necessarily represent a decrease in the amount of the analyte, but rather indicates a slowing of the increased rate. Thus, it is possible to have an increase in amount while simultaneously having a decrease in acceleration. This phenomenon is illustrated by nigericin-induced ASC-speck formation between 1 and 1.5 h. While ASC-speck formation is still rising (Fig. 1), the acceleration is decreasing (Fig. 2 F), indicating the process of ASC-speck formation has begun to change in the cell population. In a time-resolved study, quantification of acceleration may therefore more readily expose kinetic aspects of readouts. The highest acceleration rate (Fig. 2D–F) of ASC-GFP-speck formation is during either the first or second 30-min interval, indicating similar time to responsiveness with ATP, MSU and nigericin, i.e. a response regardless of trigger, although the magnitude of the response varies with trigger (Fig. 1).

### 2.3 Cytokine kinetics differ with inflammasome trigger

**Fig. 3:**
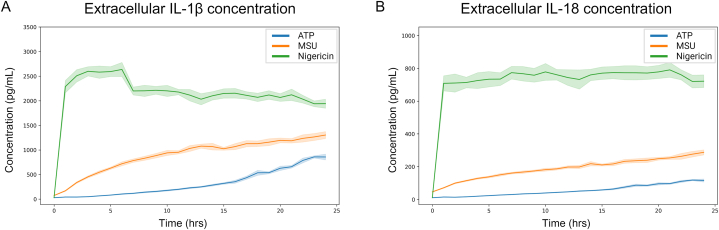
Cytokine concentration differs with inflammasome trigger. IL-1β (A) and IL-18 (B) release was quantified after triggering of inflammasome activation with either ATP, MSU or nigericin in PMA-differentiated, LPS-primed THP-1 cells. Extracellular IL-1β and IL-18 were quantified using the MSD® U-PLEX Platform. Data is displayed as mean (solid line) ± SEM (shaded area), n = 5. Created with BioRender.com.

**Fig. 4:**
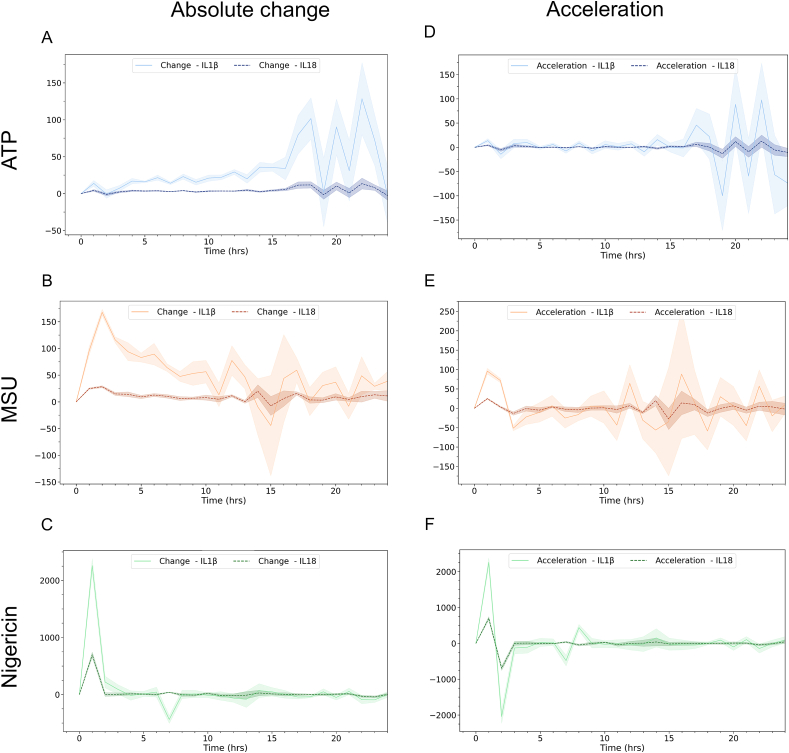
Cytokine change rates differ between triggers. Absolute change (A–C) and acceleration (D–F) of extracellular IL-1β (dashed line) and IL-18 (solid line) concentration after triggering inflammasome activation in PMA-differentiated, LPS-primed THP-1 cells with ATP (A and D), MSU (B and E) or nigericin (C and F). Extracellular IL-1β and IL-18 were quantified using the MSD® U-PLEX Platform. Data is shown as mean (solid or dashed line) ± SEM (shaded area), n = 5. Created with BioRender.com.

To investigate IL-1β and IL-18 release kinetics, extracellular cytokine concentration was quantified every hour for 24 h (Fig. 3). As with speck formation (Fig. 2), we also evaluated the absolute change and acceleration of cytokine release (Fig. 4).

The extracellular concentration of IL-1β varied after triggering inflammasome activation with either ATP, MSU or nigericin (Fig. 3A), with regards to both level and release kinetics. Triggering inflammasome activation with ATP caused a low initial release of IL-1β that gradually increased for the first 15–16 h, after which a more pronounced increase was detected (Fig. 3A). This is also reflected in the absolute change (Fig. 4A), and acceleration (Fig. 4D), where the largest increases in absolute IL-1β concentration and acceleration, respectively, start to occur 16 h after triggering with ATP. In contrast, triggering inflammasome activation with MSU leads to a more rapid release of IL-1β (Fig. 3A) with the largest increase in absolute concentration occurring at between 1 and 2 h (Fig. 4B). The highest level of acceleration, however, is prior to the largest increase in absolute concentration, between 0 and 1 h after triggering with MSU (Fig. 4E). The large variation in absolute change and acceleration observed at 15–16 h post MSU-triggering (Fig. 4 B and E) is likely due to experimental variation, as reflected in the large SEM. Nigericin was the inflammasome trigger that, among the three triggers tested, produced the largest increase in extracellular concentration (Fig. 3A), absolute change (Fig. 4C), and displayed the highest acceleration of IL-1β and IL-18 (Fig. 4F), all occurring during the first hour of the experiments. For all triggers tested, the kinetics of IL-18 release closely matched those of IL-1β release with the same trigger, although at a lower magnitude (Fig. 3, Fig. 4), displaying considerable parity between the two cytokines.

### 2.4 Temporal association between inflammasome readouts varies with trigger

**Fig. 5:**
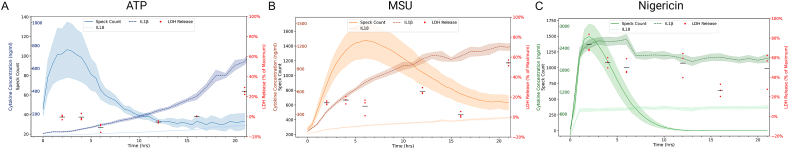
Temporal association between speck formation, cytokine release and LDH leakage varies with activating signal. After triggering NLRP3 inflammasome activation with either ATP (A), MSU (B) or nigericin (C) in PMA-differentiated, LPS-primed THP-1 cells, the temporal association of ASC-speck count, extracellular IL-1β concentration, extracellular IL-18 concentration, and extracellular LDH was assessed. ASC-GFP specks were imaged by fluorescent microscopy and automatically quantified using the Weka segmentation plugin for ImageJ. IL-1β concentration and IL-18 concentration were quantified using the MSD® U-PLEX Platform. LDH was quantified using the CyQuant™ LDH Cytotoxicity Assay Kit. Speck count, IL-1β concentration and IL-18 concentration are shown as mean (solid line, dark dashed line and light dashed line, respectively) ± SEM (shaded area). LDH is shown as individual data points (red dots) with the mean (black dash). Created with BioRender.com. (For interpretation of the references to colour in this figure legend, the reader is referred to the Web version of this article.)

The final cellular event following experimental NLRP3 inflammasome formation is pyroptosis, a caspase-1-dependent proinflammatory form of cell death associated with GSDMD-pore formation, plasma membrane rupture (PMR), often quantified as cellular leakage of LDH [8,9,11,48,49]. In order to investigate if the increases in ASC-speck formation or cytokine release were accompanied by cell lysis, LDH was measured at time points corresponding to either the highest levels of speck formation or extracellular cytokine concentration (Fig. 5). When comparing the ASC-speck formation with extracellular IL-1β and IL-18 concentration and LDH leakage, our data suggest that cytokine release is more closely connected in time to LDH than to ASC-speck formation when triggered with ATP (Fig. 5A), while triggering with MSU causes extracellular IL-1β and IL-18 to increase within the same timeframe as the increase in ASC-speck formation but seemingly not associated with LDH leakage (Fig. 5B). With regards to nigericin, the progression from triggering, through the NLRP3 inflammasome cascade to cytokine release was, with our approach, too rapid to be able to distinguish the presence of any associations (Fig. 5C).

**Fig. 6:**
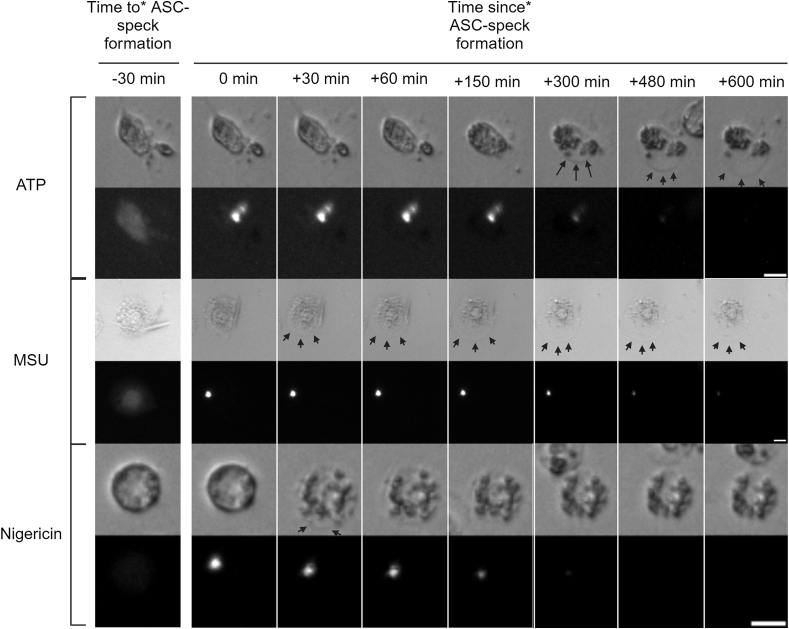
Time from ASC-speck formation to cellular swelling differs with inflammasome trigger. Temporal association between ASC-speck formation and cellular swelling in PMA-differentiated, LPS-primed THP-1-ASC-GFP cells after triggering ASC-speck formation with either ATP, MSU or nigericin. Images were obtained every 30 min for 21 h *Speck formation may have occurred at any time during the 30 min leading up to (*time to*) or since (*time since*) the indicated time point. Scale bars are 10 μm. Created with BioRender.com.

Since increases in IL-1β associate with increases in both ASC-speck formation and LDH leakage, we investigated if there was any discrepancy in association in time between ASC-speck formation and PMR. In order to assess PMR, the brightfield images taken during ASC-speck quantification were used to detect cells undergoing swelling and subsequent shrinkage, i.e. physiological changes that indicate PMR. The images indicate that ASC-speck formation precedes PMR, but the time between ASC-speck formation and PMR differ with different triggers (Fig. 6), although this requires further verification.

Assuming that during NLRP3 inflammasome activation, ASC-speck formation is a prerequisite for caspase-1 activation, and that caspase-1 activation is required for IL-1β/18 processing, GSDMD cleavage, and pyroptosis, our results suggest that either caspase-1 activation, GSDMD pore formation activation, or both can be temporally regulated. Temporal regulation of caspase-1 has been suggested previously [14]. Equally well, cytokine processing and pyroptosis may further be separate, but parallel pathways [50,51], which might explain temporal differences between ASC-speck formation and plasma membrane rupture. While LDH is a commonly used readout for pyroptosis, arguments for decoupling of cell lysis/PMR from pyroptosis have arisen [12]. Here, using a time-resolved approach, we further show that the temporal association between speck formation, swelling of cells and PMR vary with different triggers, although the order in which these events occur remains constant.

### 2.5 Experimentally induced NLRP3 inflammasome formation causes trigger-specific associations between readouts

Using our time-resolved approach, the kinetics of ASC-speck formation, cytokine release and LDH leakage, as well as potential associations between them, can be assessed in this experimental context. Furthermore, trigger-specific readout-kinetics can be identified.

ATP-activated THP-1-ASC-GFP cell populations formed a relatively low, but still significant, number of ASC-specks (Fig. 5A, Video S1). The maximum observed absolute change (i.e. when the total number of ASC-specks increases most rapidly) was at 30 min post activation (Fig. 2A), and maximum number of specks was visible at, on average, 3 h post activation (p = 0.039). This did not coincide with an increase in extracellular LDH, nor did it occur simultaneously as the highest change in IL-1β or IL-18 secretion. IL-1β secretion from the ATP-triggered cells was relatively low initially, although still significant after 1h (p = 0.01). The largest absolute changes in IL-1β occurred between 15 and 22 h after ATP triggering, despite that there was no corresponding increase in ASC-speck formation. LDH leakage did, however, increase from 0 to approximately 25 % of maximum between 16 and 21 h (Fig. 5A).

Extracellular IL-18 levels showed similar trends in ATP-triggered cells as detected with IL-1β, albeit at a lower level. Triggering with ATP caused a relatively low, but steady increase in extracellular IL-18 levels, with the highest rate of increase beginning at 15 h post activation (Fig. 3A and D), 12 h after maximum speck formation (Fig. 1, Fig. 5A), and during the period when extracellular LDH increased the most (Fig. 5A). IL-18 was significantly increased 1 h post activation (p = 0.009) and reached maximum levels at 23 h post activation (Fig. 3A).

Triggering with MSU caused a more potent but slightly more delayed ASC-speck formation (Fig. 1, Fig. 6, and Video S2) than triggering with ATP. In contrast to ATP, a closer temporal association between speck formation and cytokine release rates were observed for cells triggered with MSU. The maximum rate of speck formation was at 1.5 h after triggering with MSU, and ASC-speck count peaked after an average of 6 h (p < 0.001) (Fig. 1). Maximum rate of ASC-speck formation coincided with a slight increase in LDH leakage (Fig. 5B), but LDH levels remained constant between 2 and 6 h after triggering with MSU, during which time the rates of both ASC-speck formation and IL-1β release were relatively high (Fig. 2B and E). The maximum level of cell lysis matches the time of highest, extracellular cytokine concentration, but this increase is not accompanied by a corresponding increase in cytokine secretion. However, since cell lysis is already at 20 % of maximum after 2 h, there is presumedly a mixture of mature interleukins and their inactive pro-forms in the analyzed samples. MSU-triggered cells released IL-1β more rapidly and at higher levels than ATP-triggered cells (p = 0.001 after 1h). The highest rate of IL-1β secretion was at approximately 2 h after triggering inflammasome formation with MSU, i.e. 30 min after the highest rate of speck formation.

Nigericin lead to a high level of ASC-speck formation (Fig. 1, Fig. 5C, Video S3) with the highest rate of formation at 1 h after triggering, and maximum number of ASC-specks after an average of 2 h (p = 0.002) (Fig. 1, Fig. 5C). After 2.5–3 h of triggering inflammasome formation with nigericin, a rapid decline in GFP signal intensity led to an inability to reliably quantify ASC-GFP specks by live cell imaging past this point. One explanation might be acidification of the intracellular milieu by nigericin. The nigericin anion can bind a proton and transport it across the plasma membrane, resulting in acidification of the cytosol [52]. This decrease in intracellular pH may then negatively affect GFP fluorescence [53]. The ASC-speck formation observed prior to GFP-intensity decline was accompanied by extensive cell lysis and a rapid increase in extracellular IL-1β and IL-18 (Fig. 3, Fig. 5C), indicating a rapid progression from ASC-speck formation to plasma membrane rupture.

Interestingly, even though MSU and nigericin caused similar levels of ASC-speck formation (Fig. 1), albeit at different times post activation, there is a large difference in the amount of IL-1β and IL-18 secreted (Fig. 3). With regards to released cytokines it is not evident what proportion of mature, active cytokine to inactive pro-form is present during measurement, which is a common and major drawback of ELISA or principally similar antibody-dependent detection. Once cell lysis starts occurring, as a consequence/step of pyroptosis, inactive intracellular pro-IL-1β and pro-IL-18 also gains access to the experimental supernatant. The rapid and high levels of IL-1β observed after activation with nigericin may in part be due to the release of pro-IL-1β accumulated during priming. In order to address the potential presence of inactive pro-IL-1β, protein size needs to be analyzed using e.g. Western blot. Western blot does however come with major limitations [54], including the lack of reliable quantification. Importantly, the temporal connection between PMR and increased release of IL-1β and IL-18 requires consideration if the analysis method used cannot reliably distinguish between the inactive and active form of these cytokines.

All in all, our time-resolved approach and comparisons between inflammasome-related readouts reveal differences in cytokine secretion and cellular swelling and in PMR and LDH leakage, indicating trigger-dependent variation in time between speck formation and GSDMD pore formation and PMR, respectively.

### 2.6 Cytokine ratios vary with trigger

**Fig. 7:**
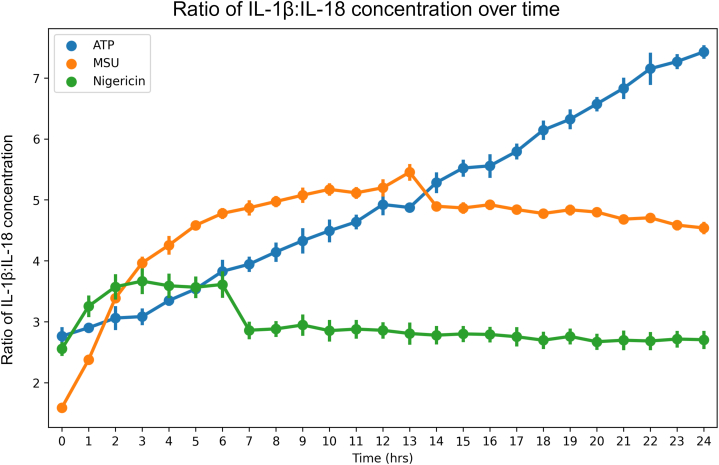
IL-1β:IL-18 ratios vary with triggers. Extracellular concentrations of IL-1β and IL-18 from PMA-differentiated, LPS- primed THP-1 cells after triggering with either ATP, MSU or nigericin were quantified using the MSD® U-PLEX Platform. The ratio IL-1β:IL-18 was calculated by dividing IL-1β concentration by IL-18 concentration. Data is shown as mean ± SEM, n = 5. Created with BioRender.com.

IL-1β and IL-18 are the two main cytokines that are directly linked to inflammasome activity, but they are described to have differing roles or contributions during the course of disease [55,56], and the interplay or ratio between the two may be as important as their amount. Distinct ratios of IL-1β and IL-18 have previously been shown to be produced during NLRP3 inflammasome activation with different triggers [57]. We therefore evaluated the ratio of IL-1β:IL-18 (Fig. 7) to investigate if there is any connection between cytokine ratio and ASC-speck formation or PMR. ATP gave the lowest increase in IL-1β:IL-18 ratio during the first 4 h, after which a continuous increase was detected for the duration of the measurements, resulting in a final IL-1β:IL-18 ratio of approximately 7:1, the highest of the investigated triggers tested (Fig. 7). Activation with MSU caused a rapid initial increase in IL-1β:IL-18 ratio (Fig. 7), which matches the rate of speck formation (Fig. 1). The highest ratio of IL-1β:IL-18 observed after MSU activation was approximately 5.5:1 at 12–13 h, after which the ratio stopped increasing. Nigericin elicited the smallest increase in cytokine ratio (ratio IL-1β:IL-18 = 3.75:1 after 3 h), which was associated to an increase in ASC-specks (Fig. 7, Fig. 1 respectively). Since IL-18 levels remain relatively unchanged after 1 h, it is possible that any effect attributed here to a changing cytokine ratio is due to a change in IL-1β concentration (Fig. 5). The consistently higher absolute rate of change of IL-1β over IL-18 (Fig. 4) further supports this. Increasing IL-1β:IL-18 ratio also occurs with MSU and ATP, the two triggers tested that induced the lowest amounts of LDH leakage. It may be that the increased survivability in the cell population permits continued cytokine production, allowing for the observed increases in IL-1β:IL-18 ratio. In contrast, the high level of cell toxicity seen with nigericin treatment may cause the stagnation in cytokine levels, as a severely decreased number of cells cannot effect a change in cytokine concentration nor, by extension, cytokine ratio. This, in combination with the fact that *IL1B,* and not *IL18*, expression is induced to high levels in THP-1 cells by LPS priming [58] may lead to the observed, continuous, rise in IL-1β:IL-18 ratio in ATP triggered cells. It also explains the relatively rapid transition to a steady ratio seen with nigericin. Further studies investigating the importance of this, and the contribution of further mechanisms to this ratio, are warranted.

### 2.7 Time from ASC-speck formation to cellular swelling varies with activating signal

The ASC-speck is often used synonymously with inflammasome activation, as its formation is seen as a prerequisite for NLRP3 inflammasome activation and function [14,59,60]. Inflammasome activation further mediates pyroptosis, a lytic cell death hallmarked by plasma membrane rupture and leakage of larger molecules such as LDH. Moreover, inflammasome-dependent plasma membrane rupture [61] is preceded by cellular swelling [49] due to GSDMD-mediated pore formation. Since GSDMD pore formation mediates both swelling and cytokine release, we re-examined the obtained microscope images to evaluate whether or not the discrepancies observed in cytokine release were also found in cellular swelling. The swelling downstream of ASC-speck formation, and the time between ASC-speck formation and swelling, varied with trigger (Fig. 6). Swelling occurred later after ASC-speck formation in ATP-triggered cells as compared to cells triggered with MSU or nigericin (Fig. 6). Cells activated with nigericin showed only minor swelling. The time to shrinkage also differed between nigericin and ATP or MSU-triggered THP-1 cells, although the time to shrinkage after triggering speck formation with ATP or MSU could not be determined. However, the fact that ATP and MSU-activated cells can remain swollen for several hours (Fig. 6), while nigericin-activated cells, in comparison, shink rapidly reveals differences in the time to shrinkage between triggers. This suggests that the time between GSDMD pore formation and PMR may vary between nigericin-triggered speck formation and the other triggers tested. Whether this is true between other triggers as well requires further study. Further investigations to determine the veracity of the observations made here are also necessary. Furthermore, the majority of cells became immobile shortly after ASC-speck formation (Video S1, S2 and S3), which indicates that cell death most often occurs shortly after speck formation regardless of PMR.

## 3 Conclusion

End-point analysis of an established readout at a specific time is and will remain a valuable tool when analyzing any number of factors and effects. However, in this is the inherent risk of missing or underestimating any dynamic capability of the pathway(s) generating the readout(s) utilized. By combining readily accessible methods, we highlight discrepancies between inflammasome readout kinetics, further illustrating the dynamic nature of inflammasome complex formation and function. Time-resolved data has several crucial advantages over end-point analyses. For example, it is capable of more accurately capturing trends in the data and, depending on the resolution of time points, can pinpoint when changes in the readout(s) occur. The use of time-resolved analysis allows for the quantification of additional metrics as compared to single, end-point analysis. The addition of metrics, such as acceleration, to the repertoire of available quantifiable metrics, allows for the pinpointing of dynamic changes in a cellular process that otherwise may have gone undiscovered. Such an approach has thereby the possibility of opening up additional avenues to investigate and to elucidate not yet understood aspects of dynamic inflammasome activation. By comparing the kinetics of readouts, assessment of temporal association between them can be used to gain insight into how different readouts, and thereby how cellular mechanisms, are interconnected.

## 4 Limitations of study

While this study highlights metrics that are not commonly used for analysis of inflammasome readouts, such as acceleration, as additional tools to further the investigation into the dynamic capability of the NLRP3 inflammasome and its associated readouts, the precise nature of events pinpointed using this approach are not determined. We have not evaluated the cause or mechanisms behind alterations in said metrics, nor have we evaluated their biological relevance. Despite the increased ability to interpret individual cell events, several readouts are still based on population level. To gain full detail into the correlation between cellular events, single cell analyses would be necessary.

The methods utilized here, while having the advantage of being easily accessible to most labs, also come with some inherent limitations. Foremost among these is the inability to distinguish between mature and immature forms of IL-1β and IL-18 by antibody-dependent methods, making accurate quantification of this inflammasome readout difficult.

Bleaching effects during fluorescence microscopy are always an important consideration, however, the bleaching of GFP observed after triggering ASC-GFP speck-formation with nigericin limits the ability to reliably quantify ASC-GFP specks considerably. Furthermore, GFP is pH sensitive and cytosolic decreases in pH may affect signal intensity post speck formation. Thus, the cause of decreases in speck number cannot be attributed to any specific mechanism, e.g. loss of fluorescence signal or speck degradation. ASC-speck formation can be assessed in fixed cells, which should avoid fluorescence-signal loss, but doing so in a time-resolved fashion is highly impractical to say the least.

In addition, ASC-specks may be released to the extracellular space and move out of focus or the field of view, or suffer a loss of signal due to decreases in pH in the cell media. While the obtained images suggest that the vast majority of specks remain in the cytosol, this is still a possibility and has an unknown impact. Due to these limitations of GFP, we focus on the dynamics leading up to and including maximum speck counts for each trigger.

## 5 Methods

### 5.1 Cell culture

THP-1-ASC-GFP cells (InvivoGen) were cultured in RPMI-1640 containing 3 mg/mL l-glutamine (Merck), 10 % heat inactivated premium grade FBS (Biowest), 10 mM HEPES (Merck), 1 mM sodium pyruvate (Merck), 4.5 mg/mL glucose (Merck) and 100 U/mL penicillin-streptomycin (Merck) in a humidified incubator at 37 °C and 5 % CO_2_. Zeocin (200 μg/mL) (InvivoGen) was added to the culture medium every other time the cells were passaged. Cell density was determined every two days and maintained between 5 × 10^5^ and 1.5 × 10^6^ cells/mL. Cells were used up to passage number ten at which time they were discarded. Cell culture was tested for mycoplasma using the Lookout® Mycoplasma PCR Detection Kit (Sigma-Aldrich) as per the manufacturers’ instructions.

For live cell imaging and LDH assay experiments, THP-1-ASC-GFP cells (3 × 10^4^ cells/well in 100 μL) were differentiated in 96-well imaging plates (Agilent Technologies). 3 × 10^4^ cells were added to each well in 100 μL of media and aspirated and re-dispensed one time to ensure a more even spread throughout the well. 100 μL of media containing 0.2 μg/mL phorbol myristate acetate (PMA) was added (final concentration 0.1 μg/mL) followed by 24 h rest at 37 °C, 5 % CO_2_ in a humidified incubator. Cells were subsequently washed three times with 37 °C media followed by 24 h of rest in fresh media.

To obtain the same number of cells/cm^2^ for cytokine measurement experiments as for live cell imaging and LDH assay experiments, 6.6 × 10^6^ cells were differentiated in 20 mL of media containing 0.1 μg/mL PMA in T75 flasks for 24 h at 37 °C, 5 % CO_2_ in a humidified incubator. Cells were washed and rested as described above prior to inflammasome activation.

To initiate NLRP3 inflammasome activation, cells were first primed with 500 ng/mL ultrapure LPS (Invivogen) for 4 h followed by activation of inflammasome complex formation by addition of either 5 mM ATP (Merck), 100 μg/mL MSU (Invivogen) or 10 μM nigericin (Invivogen). Samples for cytokine measurement were taken every hour for 24 h and stored at −20 °C.

### 5.2 Live cell imaging

Live cell imaging was performed at 37 °C, 5 % CO_2_, 80 % humidity using the EVOS™ M7000 Imaging System (Invitrogen) with On Stage Incubator equipped with an EVOS 10× fluorite objective and a GFP LED Cube. Autofocus was used in the brightfield channel at each time point to minimize photobleaching. Images were obtained every 30 min for 21 h, and at each timepoint 12 locations were imaged for each condition and replicate (n = 5 for ATP, MSU and nigericin treated cells, n = 6 for untreated controls). Images were analyzed using ImageJ [62].

### 5.3 Cytokine measurement

Samples were thawed on ice. Extracellular IL-1β and IL-18 levels were measured using the MSD® U-PLEX Platform and a QuickPlex SQ120 instrument from Meso Scale Diagnostics (Rockville, MD) according to the manufacturer's instructions. Standards were run as technical duplicates while each experimental condition was analyzed with five experimental replicates.

### 5.4 LDH quantification

LDH leakage was assessed using the CyQuant™ LDH Cytotoxicity Assay Kit (Invitrogen). The optimum cell number was determined to be between 2.5 and 5 × 10^4^ cells per well. We therefore used 3 × 10^4^ cells/well; the same number as for live cell imaging. THP-1 ASC-GFP cells were differentiated in 96-well imaging plates (Agilent Technologies), primed and activated as described above. Samples (n = 3) were assayed in technical duplicates, as were maximum and spontaneous LDH release. LDH activity was measured at 490 nm and the background at 680 nm was subtracted from the 490 nm absorbance values. Percent Cytotoxicity was calculated as $\%\;\text{cytotoxicity}=\left(\frac{\text{treated}\;\text{LDH}\;\text{activity}-\text{spontaneous}\;\text{LDH}\;\text{activity}}{\text{maximum}\;\text{LDH}\;\text{activity}-\text{spontaneous}\;\text{LDH}\;\text{activity}}\right)\times 100$.

### 5.5 Immunofluorescence staining

THP-1 ASC-GFP cells (3 × 10^4^ cells/well) were differentiated in 8-well, glass bottom μ-slide^high^ (Ibidi) and primed with 500 ng/mL of ultra-pure LPS (Invivogen). Inflammasome formation was then triggered by addition of either 5 mM ATP for 2 h, 100 μg/mL MSU for 5 h or 10 μM nigericin for 30 min Fig. S2). Cells were then washed twice with 37 °C PBS and fixed with 37 °C, 4 % PFA (Sigma-Aldrich) for 10 min at room temperature. All subsequent steps were performed at room temperature unless stated otherwise. The slides were kept in the dark during all incubation steps to minimize bleaching. Cells were washed twice with PBS for 5 min and permeabilized with 0.1 % Triton X-100, 1 % FBS in PBS for 10 min, followed by blocking with 5 % FBS in PBST for 1 h. The blocking solution was replaced with rabbit polyclonal anti-NLRP3 antibodies (Merck) diluted 1:1000 in 1 % BSA in PBST and incubated at 4 °C overnight. Cells were then washed three times with PBST for 10 min and incubated with Alexa Fluor™ 350 donkey anti-rabbit (Invitrogen) diluted 1:1000 in PBST for 1 h, before being washed three times with PBS. Cells were imaged immediately using the EVOS™ M7000 Imaging System (Invitrogen) equipped with a 40× apochromat objective (Olympus) and DAPI and GFP LED Cube.

### 5.6 Image analysis

The Trainable Weka Segmentation [63] plugin in ImageJ was trained to identify ASC-specks. Specks for the training set were manually delineated in images obtained from plates obtained over multiple different days, using all triggers evaluated. Images were then classified using the trained model. The classified images were then automatically thresholded, converted to masks and quantified using the Analyze Particles command with particle size set to 4–100 pixels and circularity set to 0.50–1.00. Random images were quantified manually to verify the accuracy of automatic quantification.

### 5.7 Data analysis

In order to analyze speck count data, the mean values of the technical replicates were calculated and used as the experimental replicate value. Maximum speck count measurements and time of maximum measurement for each sample were calculated as the mean of these maxima for each replicate. To minimize the impact of single anomalous datapoints on detection, maximum values for speck or cytokine change rate were calculated using a “sliding window” approach. For every 2-h subset of time, an average rate of speck or cytokine change was calculated, and from the 2-h window with the highest average rate of change, the individual time point with the highest rate of change across replicates was selected as the maximum value.

Rates of change for speck count and cytokine concentrations were determined at each time point by calculating the difference from the previous measurement. This produced an absolute value indicating the increase per time interval (unit/time). Acceleration was subsequently calculated at each time point by subtracting the rate of change at the previous time point from that at the current time point, resulting in the change in the rate of change (unit/time^2^).

For pairwise comparisons between measurements, Shapiro Wilks testing was performed on each dataset to determine normality, which indicated that most of the datasets were not normally distributed (p-value range of 0.002–0.77). Given the non-normality of the data and considering the small sample sizes in our datasets, we opted for a non-parametric bootstrap method for our statistical analyses. This approach is well-suited to handle non-normal distributions and is robust in scenarios involving small sample sizes. The bootstrap method has been demonstrated to outperform traditional t-tests and rank sum tests in such contexts, offering more reliable and accurate results [64]. Our implementation of the bootstrap method was modelled after Dwivedi et al.’s [64] framework, however we chose to implement this using Python instead of R.

## Data availability

The code and raw data can be accessed at https://github.com/rpotter6298/temporal_cytokine_inflammasome.

## CRediT authorship contribution statement

**Matthew Herring:** Writing – review & editing, Writing – original draft, Visualization, Validation, Software, Methodology, Investigation, Formal analysis, Data curation, Conceptualization. **Alexander Persson:** Writing – review & editing, Validation, Supervision, Methodology, Conceptualization. **Ryan Potter:** Writing – review & editing, Writing – original draft, Visualization, Validation, Software, Methodology, Formal analysis, Data curation. **Roger Karlsson:** Writing – review & editing, Supervision, Conceptualization. **Eva Särndahl:** Writing – review & editing, Validation, Supervision, Project administration, Methodology, Funding acquisition, Conceptualization. **Mikael Ejdebäck:** Writing – review & editing, Validation, Supervision, Project administration, Methodology, Funding acquisition, Conceptualization.

## Declaration of competing interest

The authors declare the following financial interests/personal relationships which may be considered as potential competing interests:co-author affiliated to Nanoxis Consulting AB - R.K. If there are other authors, they declare that they have no known competing financial interests or personal relationships that could have appeared to influence the work reported in this paper.

## Figures and Tables

**Figure:**
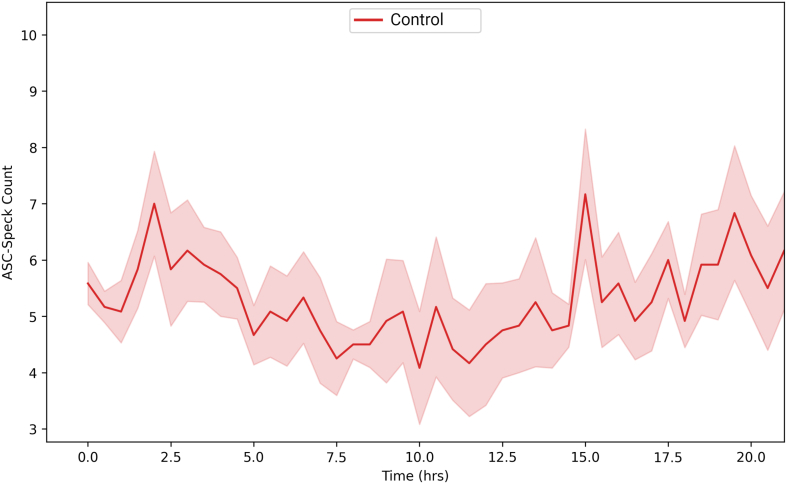
figs1. ASC-GFP speck count in untreated controls. ASC-speck count in PMA-differentiated THP-1 cells. ASC-GFP specks were imaged every 30 min for 24 hours by fluorescence microscopy and automatically quantified using the Weka segmentation plugin for ImageJ. Data are represented as mean (solid line) ± SEM (shaded area), n = 6. Created with BioRender.com.

**figs2:**
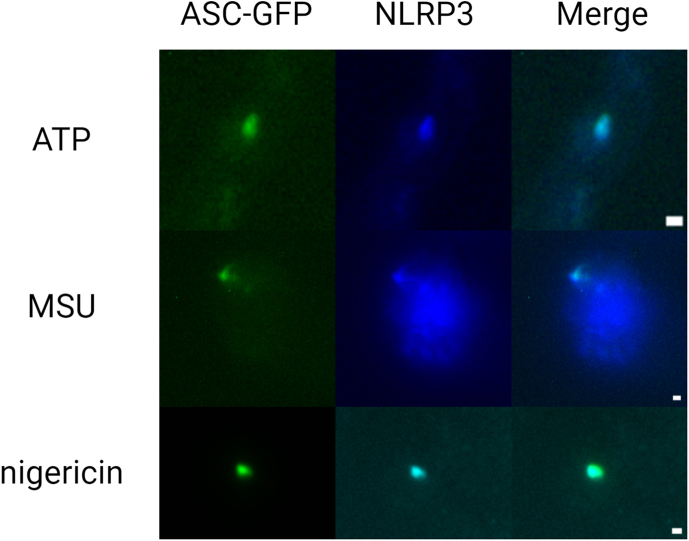
Co-localization of ASC-GFP and NLRP3. Co-localization was assessed after triggering NLRP3 inflammasome formation with either 5 mM ATP (2h), 100µg/mL MSU (5h) or 10µM nigericin (30min) in PMA-differentiated, LPS-primed THP-1-ASC-GFP cells. Cells were imaged using the EVOS™ M7000 Imaging System, 40X objective and DAPI and GFP light cube. Scale bars are 1µm. Created with BioRender.com.
